# Knittle Pressure Sensor Based on Graphene/Polyvinylidene Fluoride Nanocomposite Coated on Polyester Fabric

**DOI:** 10.3390/ma16227087

**Published:** 2023-11-08

**Authors:** Surendra Maharjan, Victor K. Samoei, Ahalapitiya H. Jayatissa, Joo-Hyong Noh, Keiichiro Sano

**Affiliations:** 1 Nanotechnology and MEMS Laboratory, Department of Mechanical, Industrial, and Manufacturing Engineering (MIME), The University of Toledo, Toledo, OH 43606, USAvictor.samoei@rockets.utoledo.edu (V.K.S.); 2 Materials & Surface Engineering Research Institute, Kanto Gakuin University, Yokohama 236-0037, Japan; noh@kanto-gakuin.ac.jp (J.-H.N.); keisano@kanto-gakuin.ac.jp (K.S.)

**Keywords:** knittle pressure sensor, graphene/PVDF nanocomposite, piezoresistive, textile, flexibility, polyester fabric

## Abstract

In this paper, a knittle pressure sensor was designed and fabricated by coating graphene/Polyvinylidene Fluoride nanocomposite on the knitted polyester substrate. The coating was carried out by a dip-coating method in a nanocomposite solution. The microstructure, surface properties and electrical properties of coated layers were investigated. The sensors were tested under the application of different pressures, and the corresponding sensor signals were analyzed in terms of resistance change. It was observed that the change in resistance was 55% kPa^−1^ with a sensitivity limit of 0.25 kPa. The sensor model was created and simulated using COMSOL Multiphysics software, and the model data were favorably compared with the experimental results. This investigation suggests that graphene-based nanocomposites can be used in knittle pressure sensor applications.

## 1. Introduction

Flexible textile sensors have gained massive momentum in research and development as a key component in wearable devices for the past two decades due to their wide range of applications in human motion detection, electronic skin, robotics, and personal health care. The flexible wearable sensor has been recognized for its unique characteristics such as enhancing breathability, softness, wearability, sensitivity, biocompatibility, and durability [1]. Textiles possess high stretchability, easy processing, low cost, and is lightweight [2]. Knitting technology has enabled them to be produced in different shapes and sizes; thus, they are the major substrate material used in wearable sensors. The sensors can be made with either conductive fibers, yarns, fabrics, or conductive materials that are coated on the raw textiles. Successful e-textile functionality is influenced by the ease of textile manufacturing, coatings, wearability, sensor reliability, and sensitivity [3].

According to the working principle, pressure sensors can be categorized into four types: piezoelectric, capacitive, piezoresistive, and triboelectric [4,5]. Piezoresistive pressure sensors have a greater potential for wearable applications among other types due to their excellent pressure tolerance, high sensitivity, wide detection range, durability, and compatibility with human skin [6,7,8,9,10,11]. Additionally, on the other hand, they possess simple data acquisition, low power input, easy fabrication, and low cost [12,13]. An external pressure in the sensor causes a change in resistance, which is sensed, analyzed, and converted into electrical signals such as resistance, current, or voltage as feedback about the information of body movement.

Wearable pressure sensors offer significant advantages for the early diagnosis of diseases and the prevention of body movement abnormalities. Specifically, wearable plantar pressure sensors can prevent foot deformities and diabetic ulcers by analyzing dynamic plantar pressure patterns [14,15]. However, detecting every human motion poses a challenge due to varying pressure levels; for instance, speaking or pulsating can exert pressure less than 1 kPa, while joint bending can generate pressures exceeding 20 kPa. Dong et al. [16] developed a wearable sensor with a hybrid 3D rGO/polyaniline sponge structure. This sensor exhibits tunable sensitivity ranging from 0.042 to 0.152 kPa^−1^ and a working range of 0–27 kPa, enabling it to detect both subtle and significant movements, such as speaking, swallowing, mouth opening, blowing, crying, and breathing.

Various studies have been conducted to investigate the impact of coatings on the durability of textiles. Wang et al. [17] demonstrated the application of epoxy coating on copper fabric tags, which exhibited excellent protection against humidity and detergents over 15 cycles. Soukup and Hamacek studied silver-coated polyamide threads used for embroidery, testing them with humidity sensors at different humidity levels. Even after 5–20 washing cycles, the sensors performed well in cyclic climate tests [18]. In Rehnby’s experiment, conductive polymers mixed with acrylic binders were applied to polyester fabrics and aged for 408 h. Subsequently, the fabrics underwent flexing, heating, rubbing, and abrasion resistance tests. The results revealed that surfaces with more active conductive polymers exhibited higher resistivity [19].

This paper introduces a knitted pressure sensor composed of a novel material. The conductive nanocomposite comprises graphene filler and a polyvinylidene fluoride (PVDF) matrix, which was coated onto a textile polyester fabric placed on a latex rubber diaphragm. The selection of the Graphene/PVDF nanocomposite material was based on its high piezoresistive behavior, as demonstrated in our previous works [20,21]. Polyester fabric was chosen due to its high melting temperature (~290 °C) and exceptionally high tensile strength (~200 MPa) among various available polymer materials. Graphene, being a 2D material with high flexibility and tensile strength (~50 GPa), is crucial for applications demanding flexible sensors such as knitted sensors. Consequently, it is believed that a novel sensor material system can be developed using graphene/polymer nanocomposites for flexible sensor applications. The paper first discusses the preparation of the graphene/PVDF nanocomposite and substrates, followed by presenting the results and simulations. The sensor’s response to various pressure inputs was recorded in terms of resistance change, and the nanocomposite material was characterized using scanning electron microscopy (SEM) images and X-ray diffraction (XRD) methods. Subsequently, the experimental results were compared with the simulation data. This sensor holds great potential for implementation in wearable flexible devices designed to detect body movements.

## 2. Sensing Mechanism

The piezoresistive pressure sensor operates based on the principle of resistance change under the application of pressure or stress. The alteration in resistance between adjacent nanoflakes is caused by three situations: changes in contact area, tunneling effects, and crack propagation. The modification in contact area between adjacent nanoflakes dominates when the applied pressure or strain is small, and electrons travel through the overlapped nanoflakes within the percolation conductive network. As the applied pressure increases, the distance between adjacent nanoflakes changes, leading to electron tunneling. This phenomenon is known as the tunneling effect, and the space between them is referred to as the tunneling distance. As this distance increases, the tunneling resistance also rises. The distance at which no electron passes through via tunneling is called the cut-off tunneling distance. The tunneling resistance between two adjacent particles can be explained using Simmons’s theory [22].

(1) $$R_{tunnel}=\frac{h^{2}d}{Ae^{2}\sqrt{2m\lambda }}\;exp\left(\;\frac{4\pi d}{h}\sqrt{2m\lambda }\right),$$

where *A*, *e*, *h*, *d*, *m*, and *λ* represent the cross-sectional area of the tunneling junction, single-electron charge, Plank’s constant, the distance between adjacent nanoflakes, the mass of an electron, and the height of energy barrier for insulators, respectively. The third one is crack propagation, which occurs when the applied pressure or strain is even higher. Initially, a crack initiates and later propagates along with time and pressure conditions. The separation of crack edges critically limits electrical conduction. The sensors developed in this paper are based on the piezoresistive mechanism.

The two important parameters of piezoresistive sensors are gauge factor (GF) and sensitivity, which can be calculated by Equations (2) and (3) [23]. The GF is determined by the change in resistance relative to the strain, whereas sensitivity is defined as the ratio of resistance change to the change in pressure.

(2) $$\mathrm{GF}=\frac{\Delta R/R_{0}}{\Delta L/L_{0}}$$

(3) $$S=\frac{\left(\Delta R/R_{0}\right)}{\Delta P}$$

## 3. Materials and Sample Preparation

A flexible conductive polymer of dimension 20 × 20 mm^2^ was designed and developed as a piezoresistive pressure sensor. The sensor was fabricated by coating conductive graphene/PVDF nanocomposite on polyester fabrics, which has a thickness in the range of 140–150 µm. The sensor was attached to a flexible rubber diaphragm (52 ± 5 µm thick) using very thin latex layer. In order to have uninterrupted sensitivity, it is important to have firm contacts between the sensor and flexible diaphragm.

The solution of nanocomposite material was synthesized via a solution-phase mixing technique. First, 1.0 g of graphite particles (1 µm thick with a purity of 99.9%) were mixed with 20 mL of acetonitrile (CH_3_CN) [24]. The large flakes of graphite were broken down by stirring the solution with the rod for a few minutes and transferred to the sonicator. The solution was sonicated 4 times in an interval of 10 min, each for 10 min to avoid aggregation of graphite particles due to temperature increase. Then, it was kept for a few hours (generally 3 to 4 h) to settle down heavy particles at the bottom, leaving behind the suspended multilayer graphene [25,26]. A total of 0.25 mg of graphene was mixed with CH_3_N in a beaker and 50.0 mg of PVDF powder (Alfa Aesar, MA, USA, Molecular Weight = 102.92 g/mol) was added to make the composite. PVDF acts as a binder in the mixture. The mixture was further sonicated twice in 10-min intervals to achieve a good solution of the nanocomposite.

**Figure 1:**
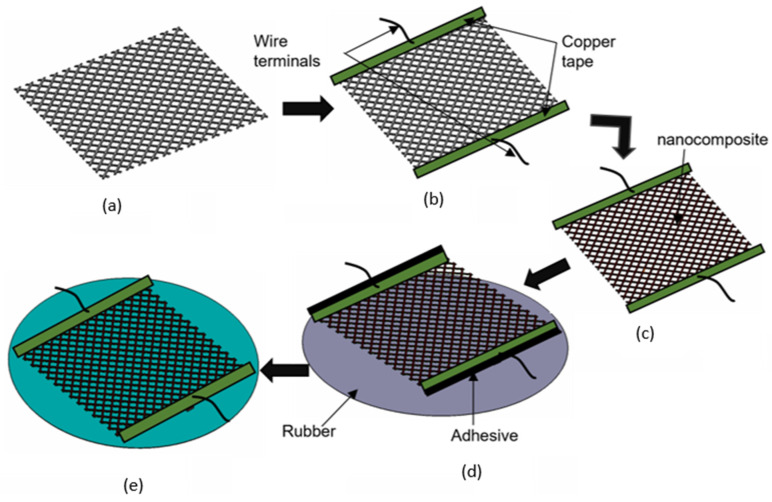
Fabrication steps of a knittle pressure sensor. (**a**) polyester fabric (**b**) adding copper tapes and wire terminal to the polyester fabric (**c**) coating the polyester fabric with the gr/PVDF nanocomposite (**d**) attaching the polyester fabric to a flexible rubber latex (**e**) final knittle sensor ready for experimental testing.

The copper tape was put at the two ends of the knitted polyester fabric and wire terminals were connected through the copper tape to measure the electrical conductivity. The fabric was cleaned with isopropanol to wash away any contaminants before applying 20–22 µm thick graphene/PVDF nanocomposite on the surface of the fabric by dip coating. The viscosity of the solution was 23.2 cP at room temperature (~25 °C) and the withdrawal speed was kept at 5 mm/s [27]. Before attaching the sensors on the rubber diaphragm, they were dried at room temperature for 24 h followed by heating at 60 °C for 2 h. Then, the coated fabric was attached to the rubber by using double face tape/adhesive at the copper tape ends. The rubber acts as a diaphragm and helps to deform knitted polyester uniformly when pressure is applied. The physical and mechanical properties of graphene, PVDF, latex rubber, and polyester fabric are presented in Table 1, and the fabrication steps of the sensor were presented in Figure 1.

The microstructure of coated films was investigated by X-ray diffraction (PANalytical X′Pert Pro MPD, Warszawa, Poland). The surface was observed via scanning electron microscopy (SEM, Hitachi S4800, Tokyo, Japan). The sensor response was measured as a change in resistance using a mega-ohm meter (Keithly 2000, Solon, OH, USA) employing a LabVIEW program and a personal computer.

## 4. Results and Discussion

### 4.1. Characterization of Graphene/PVDF Nanocomposite

**Figure 2:**
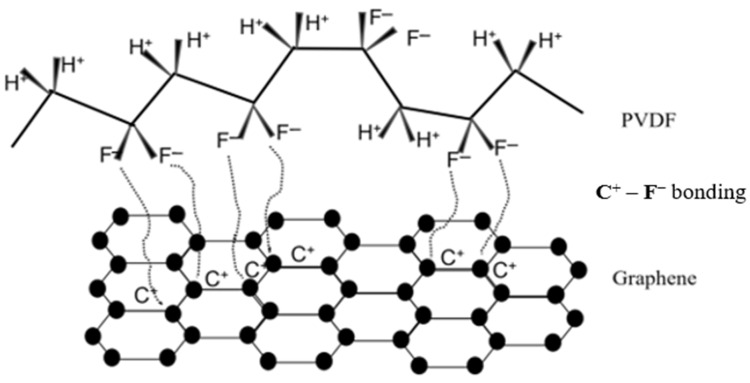
Schematic diagram of the interaction between PVDF and graphene.

**Figure 3:**
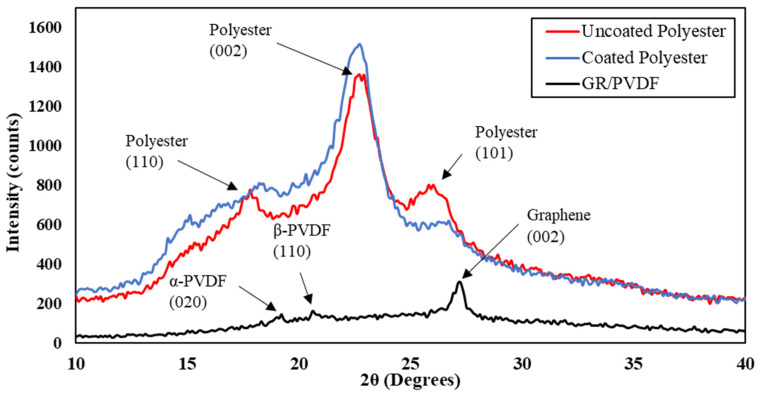
XRD pattern of graphene/PVDF nanocomposite.

**Figure 4:**
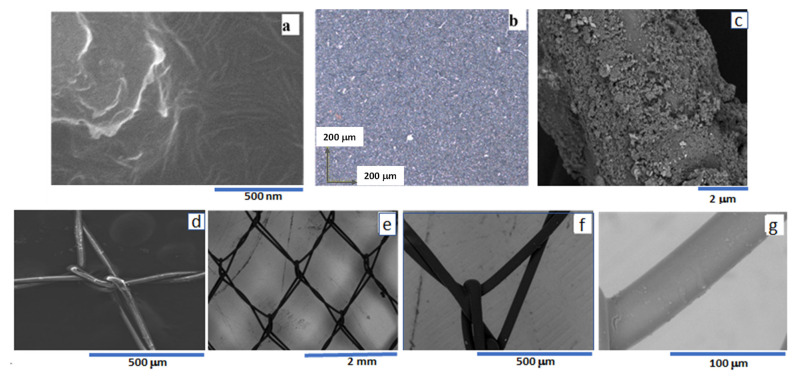
(**a**) SEM image of the nanocomposite coated by spin coating [28], (**b**) optical image of the nanocomposite, (**c**) SEM image of nanocomposite coated on a polyester fiber (high resolution, (**d**) SEM image of uncoated polyester fabric (5 nm of gold were coated for SEM imaging purpose), and (**e**–**g**) SEM image of nanocomposite-coated polyester fabric at a different scale.

(a) **Bonding between PVDF and graphene:** The bonding between PVDF and graphene is due to the presence of electron-rich benzene rings in graphene and electron-poor fluorine atoms. This polar ionic bond between carbon and fluorine atoms (C^+^–F^−^) is an attractive interaction that holds the nanocomposite together. Thus, PVDF acts as a good binder in the formation of conductive graphene/PVDF nanocomposite [28]. PVDF is a versatile material in nanocomposites, especially for sensors and flexible electronics. Its inherent piezoelectricity enables the conversion of mechanical stress into electrical signals, crucial for sensors. PVDF’s flexibility and chemical stability allow it to be molded into various forms and resist harsh environments, making it ideal for wearable devices. Moreover, its compatibility with diverse nanoparticles enhances its mechanical and electrical properties, while its biocompatibility suits medical applications. PVDF’s thermal stability and cost-effectiveness further enhance its appeal, making it pivotal in the development of advanced nanocomposite technologies, particularly in sensors and flexible electronics. The schematic of bonding between carbon and fluorine atoms is presented in Figure 2.
(b) **X-Ray Diffraction (XRD):** The XRD pattern of uncoated polyester, coated polyester, and graphene/PVDF nanocomposite is shown in Figure 3. The three main characteristic peaks of uncoated polyester in the diffraction pattern were seen at 2θ angles of 17.9° (110), 22.7° (002), and 26.1° (101). In coated polyester, the characteristic peaks were found at 2θ angles of 18.4°, 22.9°, and 26.7°. The intensities of the peaks were increased, broadened, and shifted which can be attributed, respectively, to the possible increase in the crystal phase, the formation of nanocrystalline phases, and the surface stress of coated nanocomposite on the polyester fabric. These peak positions agree with the peaks reported in other research papers [21,29,30,31]. Muthukumar et al. reported similar XRD patterns for polyester and polyester polyaniline (PANI) fabrics [32].
  The nanocomposite was separately examined by XRD. PVDF is a semi-crystalline polymer that can exhibit α, β, γ, and δ crystallographic forms. The characteristic peak of the α-phase of PVDF was observed at 2θ angles of 19.2° (002). The characteristic peak at the 2θ angle of 20.48° (110) indicates the presence of β-phase of PVDF, which is desirable for piezoelectric characteristics for the polymer and can be implemented in developing piezoelectric-based sensors. Natural graphite is a highly crystalline material that displays a very sharp peak [29]. However, the peak at a 2θ angle of 27.2° (002) is less intense, indicating the proper exfoliation of graphite into graphene.

(c) **Scanning Electron Microscopy (SEM) and Optical Microscopy (OM):** The morphology of nanocomposite and coated polyester fabric were analyzed under SEM and OM as shown in Figure 4. The images of nanocomposite film in Figure 4a,b do not show any aggregation of graphene and PVDF, indicating homogeneous dispersion of PVDF into graphene matrix and formation of graphene layers. The nanocomposite film in the polyester fabric was evenly coated without any damage as shown in Figure 4c–e. This result suggests that graphene/PVDF nanocomposite film can be coated on polyester fabric for the fabrication of textile sensors. Manasoglu et al. [33] had also shown the homogeneous coating of graphite nano-powder on polyester fabric to study electrical resistivity and the thermal conductivity properties.

### 4.2. Measurement of Sensor Response

**Figure 5:**
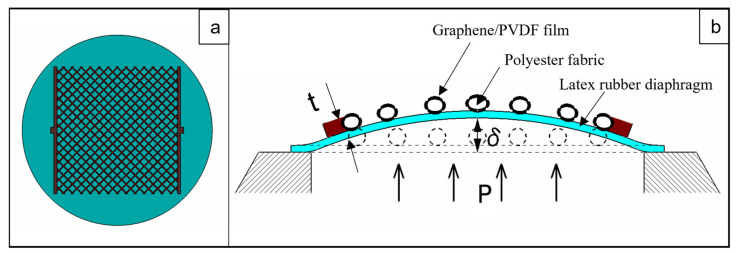
Schematic of (**a**) top view of the sensor, and (**b**) section view of deflection under applied pressure.

The main application of this sensor is to measure applied pressure based on the piezoresistive principle. The strain gauge factor is estimated to be 1.9 ± 0.2 for graphene/PVDF materials used in this research. The sensor was subjected to various pressures and the corresponding change in resistance of the conductive fabric was recorded. Further, the test was conducted for repeatability and reproducibility of the sensor by loading and unloading for various cycles. As shown in Figure 5, when the pressure is applied, the diaphragm along with the polyester fabric and nanocomposite attached to it stretches out. The conductive nanocomposite particles separate apart decreasing the number of charge carriers and conductive paths. Thus, the resistance increases. The resistance comes to its original state after unloading as the nanocomposite and substrate regain their position since the operation is in the elastic region. The mean response with time by loading and unloading of the sensor can be calculated using Equation (4),

(4) $$\frac{\Delta R}{R}\left(\%\right)=\frac{\left(R_{loading}-R_{unloading}\right)}{R_{unloading}}\times 100\%.$$

**Figure 6:**
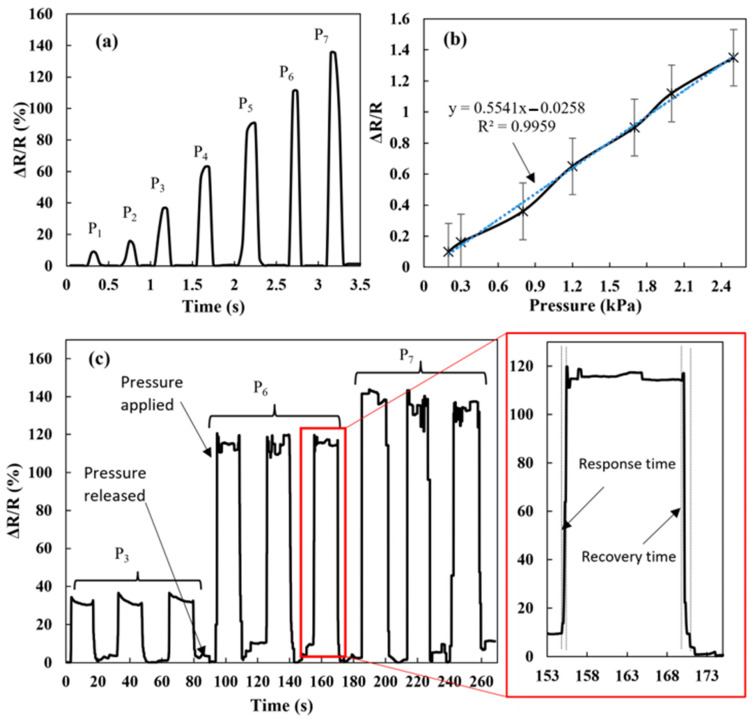
(**a**,**b**) Time response in terms of resistance changes at different pressures, and (**c**) repeatability test at three different pressures, the red square indicates a zoomed section of one of the repeatability test to clearly show the response and recovery time. In (**a**), P_1_, P_2_, P_3_, P_4_, P_5_, P_6_ and P_7_ correspond to 0.25, 0.30, 0.80, 1.2, 1.7, 1.9 and 2.5 kPa, respectively.

Figure 6 shows the time response curve in terms of resistance change and repeatable pattern during loading and unloading at three representative pressure P_3_, P_6_, and P_7._ The response versus pressure curve is an almost straight line, indicating the linear response of the sensor with pressure. The maximum applied pressure of 2.5 kPa has a response of 135%. Linearity is the main characteristic required in sensor development. The slope gives the sensitivity of the sensor, which is about 0.55 kPa^−1^. A piezoresistive sensor made up of a graphene composite sponge has reported a high sensitivity of 0.26 kPa^−1^ in a limited pressure sensing range (<2 kPa) [30]. Therefore, this sensor has potential application in wearable technology to detect body movement in a low-pressure range. The average response and recovery time of the proposed sensor were calculated as 300 ms and 700 ms, respectively. However, this time also includes pressure buildup time in the testing system; hence, the estimation of response time from the response curve does not give the actual response time of the sensor.

### 4.3. Temperature Dependence on Electrical Conductivity

**Figure 7:**
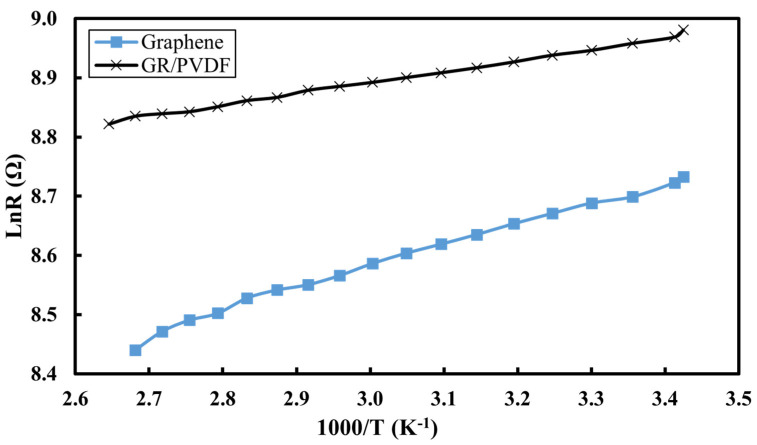
Arrhenius plot of electrical conductivity of Graphene/PVDF nanocomposite with temperature.

The graphene/PVDF solution was coated on the glass slide and connected to two terminals to measure the resistance. The sample was heated from room temperature to 150 °C and the resistance was recorded for every 5 °C change. Similarly, the process was repeated for the graphene-only sample. The average value of resistance for heating and cooling was evaluated and then plotted in the graph of ln(R) versus 1000/T, which is shown in Figure 7. The graph follows Equation (5), showing a decrease in resistance with an increase in temperature.

(5) $$\mathrm{lnR}=\left(-\frac{E_{a}}{K_{b}}\right)*\frac{1}{T}+{\mathrm{lnR}}_{0},$$

where E_a_, K_b_, R_0_, and R are the average thermal activation energy of conductivity, Boltzmann constant, inverse of cohesion factor, and resistance at T, respectively. The rise in electrical conductivity with an increase in temperature can be best described as the increase in density of the charge carrier. Thus, the nature of the graph depicts the semiconductor behavior of the nanocomposite. The E_a_ values estimated for graphene and nanocomposites are 0.026 and 0.02 eV, respectively.

### 4.4. I-V Characteristic Curve of Graphene/PVDF Nanocomposite

**Figure 8:**
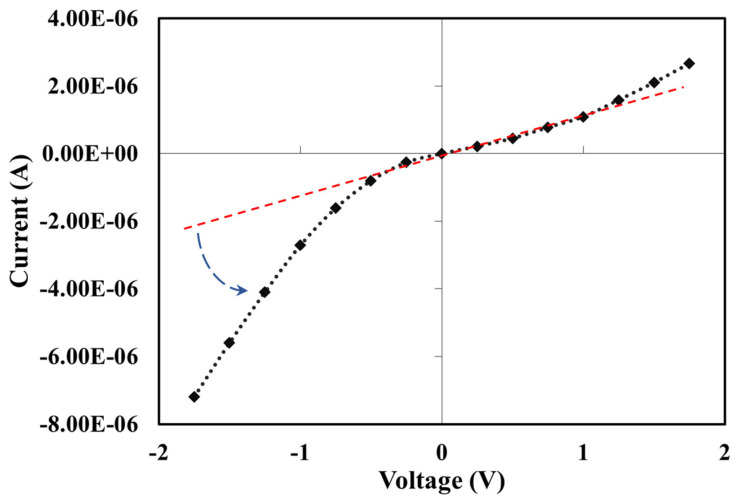
Current-voltage (I-V) characteristics of Graphene/PVDF nanocomposite with copper contacts. (Black line indicates as measured I-V data and red line indicates I-V characteristics assuming ohmic contacts).

Figure 8 shows the current-voltage (I-V) characteristic of the sensor using copper contact terminals. The graph shows a non-linear I-V characteristic for the graphene resistor. The shift of current at reverse bias (-voltage region) from the linearity is higher than the current at forward bias. This phenomenon indicated that the composite material and metal contact are not ohmic, but it has a metal/p-type Schottky junction. The Hall effect measurements were conducted for graphene/PVDF nanocomposites, and it was found that the composite is a p-type material at room temperature. The estimated electrical properties are listed in Table 2. It is important to note that these properties can be drastically changed with the change in graphene content. We believe that the p-type conduction is attributed to the oxygen adsorption in graphene [34,35].

## 5. Finite Element Analysis (FEA)

The simulation model of the sensor was prepared similar to Figure 5a. The model consists of a thin film of nanocomposite having a thickness of 20 ± 2 µm, fixed on the top of polyester fabric. Two opposite edges of the polyester fiber are fixed at latex rubber. The rubber helps to deform polyester fiber uniformly when the pressure is applied. The model was simulated in COMSOL Multiphysics, and the results were analyzed.

**Figure 9:**
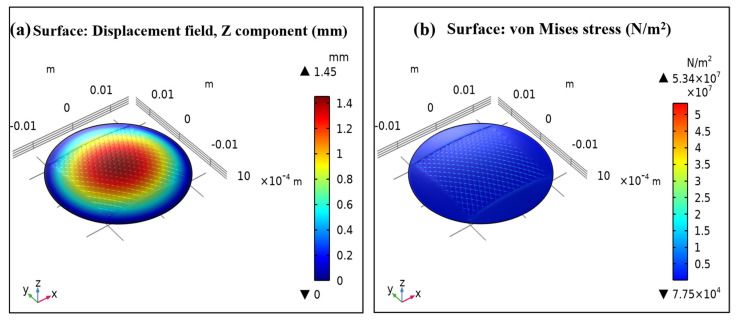
(**a**) Displacement distribution, and (**b**) stress distribution on the sensor at 2.5 kPa.

Figure 9a,b present the plots for displacement distribution in Z–the direction and Von mises stress distribution. For mechanical analysis, 2.5 kPa pressure was applied and the corresponding displacement and Von Mises stress were observed. Table 3 gives the summary of the FEA parameters used in this simulation. Uniform pressure (N/m^2^) was applied in the z-direction {0, 0, Pz}. A total of 2.5 kPa pressure with each increment of 0.25 kPa and a total of 10 iterations was applied. Table 2 gives a summary of the parameters used for this analysis. The maximum displacement and Von Mises stress on the sensor were observed at 1.5 mm and 53.4 MPa, respectively. Maximum stress was developed at the corner of the square fabric, which has less effect on the failure of the sensor, and it is even less than the yield strength (54 MPa), indicating the safe design of the sensor. In other words, the sensor does not deform permanently at this pressure application.

**Figure 10:**
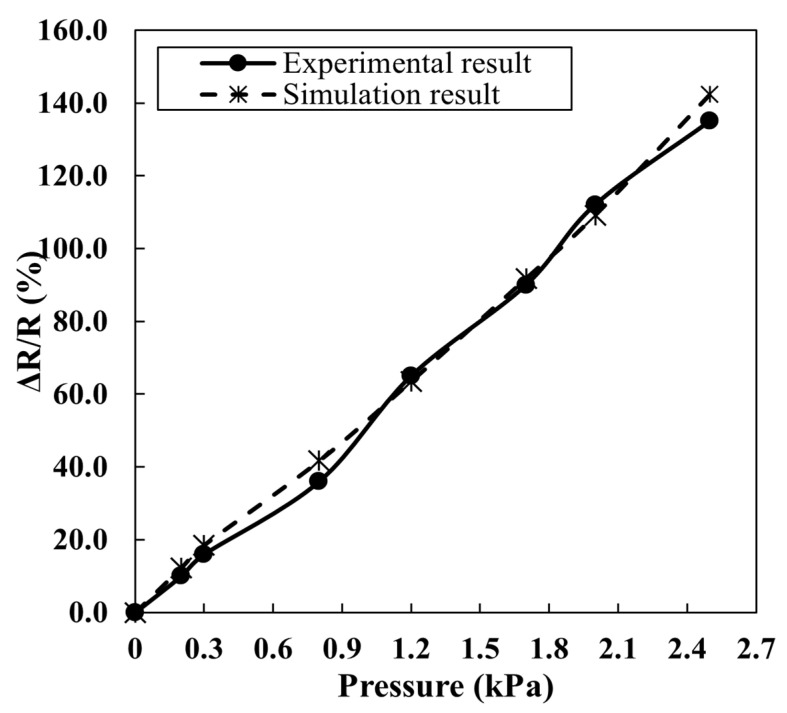
Measured (continuous line) and simulated (broken line) sensor response as a function of pressure.

For the electrical analysis, the pressure varied from 0.25 to 2.5 kPa and the corresponding resistance was recorded. The response graph was plotted against pressure and compared with the experimental result. As shown in Figure 10, the response curves for both the experimental and simulation result were close, indicating the validation of the experimental data. The correlation factor between experimental and simulated data is 98.7%, suggesting that the proposed model reasonably explains the piezoresistive sensor mechanism. The linearity of the curve helps to calibrate the sensor more accurately.

## 6. Conclusions

A flexible knitted pressure sensor was successfully developed utilizing a graphene/PVDF nanocomposite on a polyester substrate. PVDF served as a robust mechanical binder on the graphene matrix, forming strong ionic bonds between carbon (C) and fluorine (F) atoms. Applying pressure to the diaphragm surface enabled the recording of corresponding loading and unloading resistances, allowing for calibration in terms of resistance change. Remarkably, the proposed sensor demonstrated an average response time of 300 ms and a recovery time of 700 ms, showcasing its impressive performance in sensor applications. The sensor exhibited a sensitivity of 55% kPa^−1^ within the pressure range of 0–2.5 kPa, translating to a sensor response (ΔR/R) of 0.55 when subjected to 1 kPa pressure. Furthermore, the sensor’s behavior was accurately simulated using Comsol Multiphysics, with the response curve closely aligning with experimental data. Adhering to the Von Mises criteria, the sensor proved to be secure from failure within the applied pressure range. These results underscore the substantial potential of the proposed sensor for integration into wearable and flexible electronic devices, contributing significantly to the rapid advancements in smart technology.

## Figures and Tables

**Table 1:** Physical properties of graphene, PVDF, and substrates [27,28,29,30,31] together with dimensions of sensors fabricated in this investigation.

Properties	Graphene	PVDF	Latex Rubber	Polyester Fabric
Density (kg/m^3^)	2250	1780	970	1380
Melting Point (°C)	3650	155–160	180	295
Resistivity (Ω⋅cm)	10^−6^	2 × 10^14^	–	–
Electrical Conductivity (S/cm)	6000	–	–	–
Young’s Modulus (GPa)	1000	2.45	0.1	0.92
Yield strength/Tensile strength (MPa)	240–280	25–60	10–20	53.8–265
Poisson’s Ratio	0.149	0.34	0.49	0.43
Dimensions used in sensor (µm)	*t:20 µm *A:20 mm × 20 mm (Thin film Coating)		*t:52 µm *r:15 mm	*t:140 µm *A:20 mm × 20 mm

*t, A, and r, are thickness, area, and radius, respectively.

**Table 2:** Electrical properties of Graphene/PVDF nanocomposites used in this investigation.

Property	Value
Career density (n), cm^−3^	2.63 × 10^16^
Hall voltage (V_H_), V	0.022
Mobility (µ), cm^2^/(v⋅s)	156.33
Conduction type	P-type

**Table 3:** Input parameters for the FEA.

Name	Expression	Value	Description
Pz	2500 [N/m^2^]	2500 N/m^2^	Force per unit volume
rho_f	1780 [kg/m^3^]	1780 kg/m^3^	PVDF density
rho_m	2250 [kg/m^3^]	2250 kg/m^3^	Graphene density
rho_c	rho_f*V_f + rho_m*V_m	2240.8 kg/m^3^	Composite density
V_f	0.0196	0.0196	Fiber volume fraction
V_m	1 − V_f	0.9804	Resin volume of fraction
E_m	1 [TPa]	1 × 10^12^ Pa	Graphene Young’s modulus
nu_c	0.19	0.19	Composite Poisson’s ration
nn	2.5578 × 10^12^ [1/mm^3^]	2.5578 × 10^21^/m^3^	The number density of graphene
sigma0	3 × 10^3^ [S/cm]	3 × 10^5^ S/m	Electrical conductivity of Graphene

## Data Availability

All data is reported in this paper.
